# Systematic pan-cancer analyses of the potential function of the Golgi scaffold protein PAQR3

**DOI:** 10.1038/s41598-024-53489-z

**Published:** 2024-02-06

**Authors:** Zhe-Nan Ling, Lian-Lian Hong, Jian Wu, Zhi-Qiang Ling

**Affiliations:** 1 Division of Hepatobiliary and Pancreatic Surgery, Department of Surgery, The First Affiliated Hospital, Zhejiang University School of Medicine, 79 Qingchun Road, Hangzhou, 310003 Zhejiang People’s Republic of China; 2 NHC Key Laboratory of Combined Multi-organ Transplantation, Hangzhou, Zhejiang People’s Republic of China; 3 Key Laboratory of the Diagnosis and Treatment of Organ Transplantation, Research Unit of Collaborative Diagnosis and Treatment for Hepatobiliary and Pancreatic Cancer, Chinese Academy of Medical Sciences (2019RU019), Hangzhou, Zhejiang People’s Republic of China; 4 Key Laboratory of Organ Transplantation, Research Center for Diagnosis and Treatment of Hepatobiliary Diseases, Hangzhou, Zhejiang People’s Republic of China; 5 Zhejiang Cancer Institute, Zhejiang Cancer Hospital, Hangzhou, 310022 Zhejiang China; 6 Hangzhou Institute of Medicine (HIM), Chinese Academy of Sciences, Hangzhou, 310018 Zhejiang China

**Keywords:** Progestin and adipoQ receptor3 (PAQR3), Chemotherapeutic potential, Tumor diagnosis, Prognostic value, Pan-cancer tissues, Cancer, Molecular biology, Molecular medicine, Oncology

## Abstract

Progesterone and AdipoQ Receptor 3 (PAQR3) is a member of the AdipoQ receptor. Our previous studies have found that PAQR3 plays a role as a candidate inhibitor in cardiac adenocarcinoma, breast cancer, gastric cancer and colorectal cancer, but the systematic analysis of PAQR3 in tumors is currently lacking. The objective of this study was to investigate the prognostic and therapeutic value of PAQR3 in 31 tumors. Through the analysis of TCGA, UALCAN, GEO, GEPIA2, TIMER, Kaplan–Meier plotter, TISIDB and other databases, it was found that the expression level of PAQR3 changed significantly in different tumor types, and the expression level of Neuroblastoma was very high. And the level of Prostate adenocarcinoma is low. In addition, the expression level of PAQR3 in Cholangiocarcinoma, Esophageal carcinoma, Head and neck squamous carcinoma, Liver Hepatocellular Carcinoma, Lung Adenocarcinoma and Lung squamous cell carcinoma was significantly higher than that in normal tissues. However, the expression level of PAQR3 in Breast Cancer, Kidney Renal Clear Cell Carcinoma, Kidney renal papillary cell carcinoma, Prostate Adenocarcinoma, Rectum Adenocarcinoma, Thyroid Cancer and Uterine Corpus Endometrial Carcinoma was lower than that in normal tissues. Subsequently, we explored the value of PAQR3 as a prognostic indicator of cancer. In Acute Myeloid Leukemia, Lower-grade Glioma and Glioblastoma, Pediatric Low-grade Gliomas, Kidney Chromophobe, and Thyroid Cancer, PAQR3 expression was positively correlated with OS and DSS, while in Rectum Adenocarcinoma, PAQR3 expression was negatively correlated with OS. PAQR3 high expression group Lower-grade Glioma and Glioblastoma, Pediatric Low-grade Gliomas, Uveal Melanoma, Kidney Chromophobe and DFI were positively correlated. PAQR3 can be used as a risk factor for the prognosis of multiple tumors. Then, we discussed the correlation between PAQR3 and immunology, and found that PAQR3 has a wide range of mutations in various tumor types, the most common mutation type is missense mutation, and common mutation types also include amplification, depth deletion, splicing, truncation and structural variation. Among the tumor samples with PAQR3 alterations, mutation occurred in all tumor samples except prostate adenocarcinoma and adrenal cortical carcinoma, head and neck squamous cell carcinoma, brain low-grade glioma, and kidney clear cell carcinoma, while esophageal adenocarcinoma had the highest total alteration frequency. PAQR3 was strongly associated with CNV in 18 tumors, particularly in Ovarian cancer, Lung squamous cell carcinoma, and Adenoid cystic carcinoma. On the other hand, PAQR3 has a higher SNV frequency in Uterine Corpus Endometrial Carcinoma, Skin Cutaneous Melanoma and Lung Adenocarcinoma, among which Uterine Corpus Endometrial Carcinoma has the highest SNV frequency. These results showed that PAQR3 expression levels were significantly correlated with tumor mutation load, microsatellite instability, neoantigens, and purity. In summary, PAQR3 can affect the tumor microenvironment and has potential for chemotherapy. Finally, we investigated the role of PAQR3 in tumor resistance and found that the expression of PAQR3 affects the efficacy of multiple chemotherapy drugs. Based on these studies, we found that PAQR3 plays an important role in cancer and has potential in tumor diagnosis and prognosis.

## Introduction

Cancer is the leading cause of death worldwide, with nearly 10 million deaths due to cancer in 2020. Many tumors are curable when detected early and treated effectively^1^. Each cancer type requires a specific treatment plan, so the right diagnosis and prompt treatment is very crucial for cancer. Treatment for tumors includes surgery, radiotherapy, and systemic therapy (chemotherapy, targeted biotherapy, hormone therapy). However, radiotherapy and chemotherapy are not always effective, and the tumor’s tolerance to treatment seriously affects the course of treatment, and the serious side effects associated with treatment also reduce the quality of life of patients. At present, immunotherapy as a new tumor treatment method has made excellent progress in clinical practice^2^. However, the response to treatment is still limited to a small number of patients, so the link between genome, epigenome and immune elements in tumor needs to be further studied.

PAQR3 is a member of the progestin and adipoQ receptor (PAQR) family. It is similar to G protein in structure and has seven transmembrane structures. It is a kind of receptor molecule distributed in Golgi apparatus. Current studies on PAQR3 have found that this molecule plays a role as a tumor inhibitor in non-small cell lung cancer (NSCLC) by blocking the interaction between BECN1 and the activated form of EGFR, enhancing the autophagy induced by EGFR inhibitor erlotinib, and inhibiting the NF-KB/p53/Bax axis^3–7^. In a study on the mechanism of diabetic wound production, PAQR3 silencing was found to promote angiogenesis and accelerate skin wound repair in diabetic mice and polarization of M2 macrophages with increased expression level of PPAR γ (peroxisome proliferation-activated receptor γ). Further studies showed that, PAQR3 in macrophages influences the polarization of macrophages by regulating PPAR γ protein stability through the ubiquity-proteasome pathway^4^. In addition, PAQR3 negatively regulates the PI3K/AKT signaling pathway, as a key regulator of inflammation and metabolism, as well as participating in the regulation of cell death, which has been reported in studies on the mechanism of neuron apoptosis during ischemia reperfusion and metabolic diseases such as diabetes^5,6^. Current studies on PAQR3 have found that this molecule acts as a tumor suppressor in NSCLC, BRCA, PA (pulmonary adenocarcinoma) and other tumors to inhibit tumor proliferation, promote tumor cell apoptosis and sensitivity to targeted drugs, and is related to the clinical stage and prognosis of the disease^3,7–9^. Based on the current research status of PAQR3, we speculate that this molecule can be used as a potential molecular marker for prognosis and treatment.

At present, there are limitations in the study of PAQR3. If we want to explore whether the role of PAQR3 in various cancers is universal and which tumors can be used as prognostic or therapeutic markers, we still need to analyze the correlation between PAQR3 expression at the pan-cancer level, prognostic indicators, and tumor microenvironment. This research makes a systematic and comprehensive study of the correlation between PAQR3 expression profile, prognostic significance, and tumor microenvironment by bioinformatics methods. This study will provide a new perspective for the research of PAQR3 in cancer and a new idea for the early diagnosis and treatment of cancer.

## Methods and materials

### Gene expression analysis

For the expression difference analysis of PAQR3, we used CCLE, TIMER, and GEPIA2 to investigate its expression differences in pan-cancer from the perspective of cell lines and tissues.

TIMER is based on the TCGA database, 6 kinds of tumors infiltrating immune subgroups from 32 cancer types were analyzed and calculated. The functional modules of TIMER include somatic Mutation (Mutation), DNA somatic cell copy number alterations (SCNA), differential gene expression analysis (DiffEXP), and gene–gene correlation analysis (Correlation)^10^.

The GEPIA2 website is a resource for gene expression analysis of tumor and normal samples based on the TCGA and GTEx databases. The tool supports quantification of gene expression from the gene level to the transcriptional level and comparison between specific cancer subtypes^11^.

CCLE database (https://portals.broadinstitute org/CCLE/about) is applied to analyze the expression level of PAQR3 pan-cancer cell lines^12^.

### DNA promoter methylation analysis

UALCAN analyzed gene expression differences between tumors and normal samples based on TCGA RNA-Seq and 31 kinds of tumors clinical data, and estimated the impact of gene expression levels and clinicopathological features on patient survival by individual cancer stage, tumor grade, ethnicity, or other clinical history characteristics^13^.

For the expression difference analysis of PAQR3, we used CCLE, TIMER, and GEPIA2 to investigate its expression differences in pan-cancer from the perspective of cell lines and tissues.

### Clinical prognoses analysis

PrognoScan (http://dna00.Bio.Kyutech.Ac.Jp/PrognoScan/index. The HTML) is applied to the analysis of the correlation between PAQR3 gene expression and clinical prognostic indexes OS and DFS.

Kaplan–Meier plotter analyzed the correlation between PAQR3 expression level and OS in pan-cancer^14^.

Log rank test was used to analyze the survival difference, and *P* < 0.05 was the significance index.

Sangerbox is an online analysis tool based on TCGA and GTEx databases. The tool was used to analyze the correlation between PAQR3 and tumor mutation burden, microsatellite instability, neoantigens and purity. Using the TCGA dataset through SangerBox, we investigated the relationship between CCNE1 mRNA expression levels and tumor mutation burden (TMB), microsatellite instability (MSI), neoantigens, and immune checkpoint (ICP) genes in cancer. Using the TCGA dataset through SangerBox, we investigated the relationship between CCNE1 mRNA expression levels and tumor mutation burden (TMB), microsatellite instability (MSI), neoantigens, and immune checkpoint (ICP) genes in cancer.

### Immunophenotype association analysis

TISIDB is an online tool containing immunological data on a variety of tumor types, using high-volume screening and genomic analysis to identify genes associated with T-cell-mediated killing and immunotherapy in tumor cells^15^. In this study, TISIDB was used to analyze the correlation between PAQR3 and immune subtypes in pan-cancer. *P* < 0.05 was used as the data significance cut-off line.

### Gene mutation analysis

Mutation analysis of PAQR3 was performed using the cBioPortal database to calculate its mutation frequency and the GSCA to calculate the correlations between CNV, SNV, and various tumors.

cBioPortal (https://www.cbioportal.org) provides a visual and multi-angle analysis of tumor genome data in the TCGA database^16^.

GSCA (http://bioinfo.life.hust.edu.cn/GSCA) is used for cancer gene set analysis at the genomic, drug genomic, and immune genomic levels. This tool can analyze the relationship between immune infiltration and gene expression and genomic variation, and between gene set expression/mutation and clinical outcome^17^. Mutation analysis of PAQR3 was performed using the cBioPortal database to calculate its mutation frequency and the GSCA to calculate the correlations between CNV, SNV, and various tumors.

### Pharmacological analysis

Cellminer contains new databases and analytical tools related to whole exome sequencing and protein expression, which is to integrate and analyze drug-related data for 60 tumor classes^18^. In this study, Cellminer was used to analyze the correlation between drug data and PAQR3 expression level in 60 kinds of tumor cells. The correlation between PAQR3 and the drug IC50 is defined by the R package “impute,” “limma,” “ggplot2.”

### Ethics declarations

The studies involving human participants were reviewed and approved by the Institutional Review Board of Zhejiang Cancer Hospital (IRB-2023-245, Scientific research). All data analysis were performed in accordance with relevant guidelines and regulations.

## Results

### The expression level of PAQR3 in pan-cancer was significantly different

**Figure 1:**
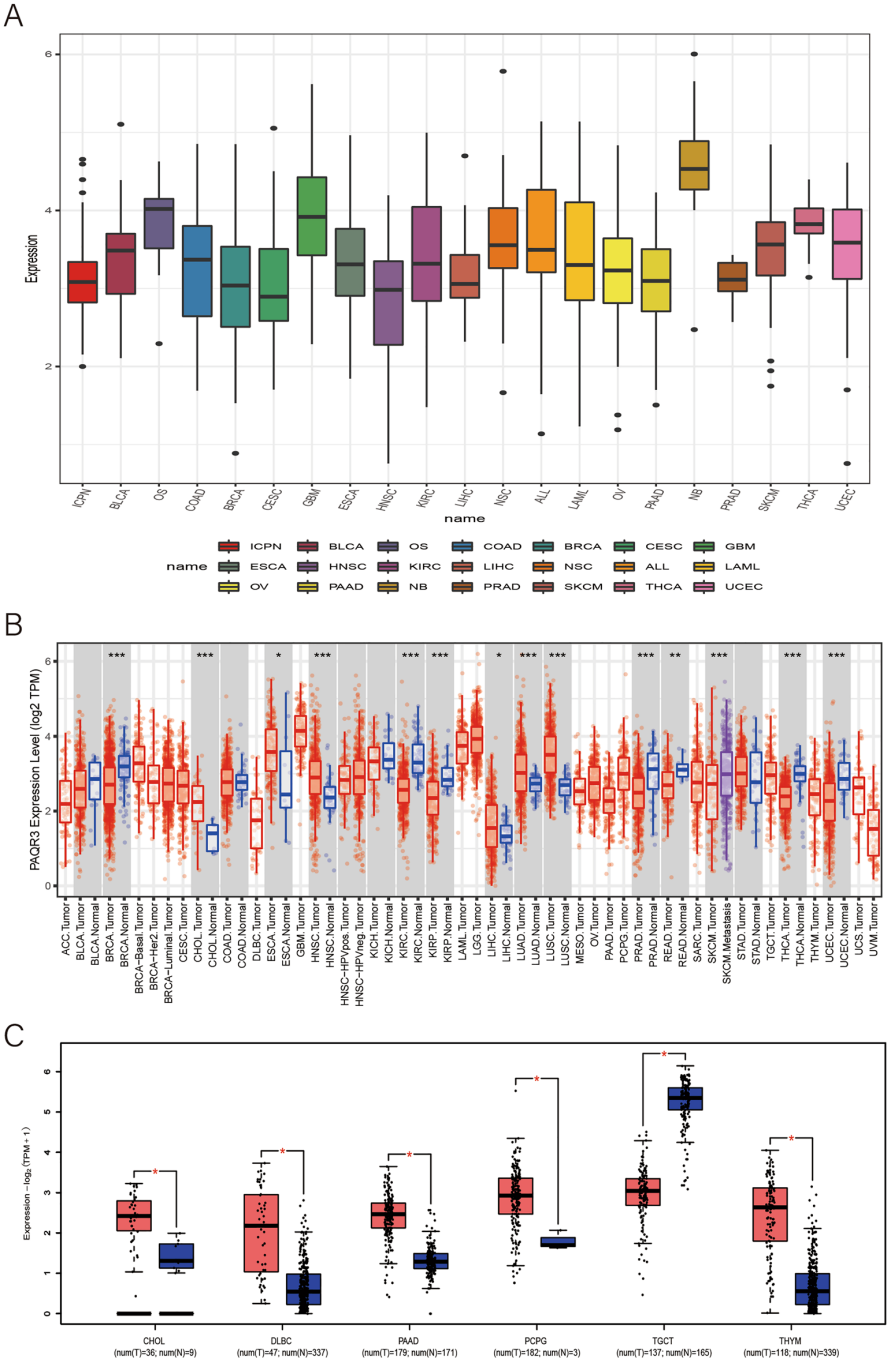
Expression level of PAQR3 in human tumors. PAQR3 expression levels in different tumors. (**A**) The expression level of PAQR3 was significantly elevated in NB and particularly low in PRAD. (**B**) The expression level of PAQR3 in tumor tissues and normal tissues in the TCGA database, the expression level of PAQR3 in 6 types of tumors was significantly higher than that in normal tissues, and the expression level of PAQR3 in 7 types of tumors was significantly lower than that in normal tissues. (*P < 0.05, **P < 0.01, ***P < 0.001). (**C**) Expression level of PAQR3 in tumor tissue and normal tissue in GEPIA2 database, red representing tumor tissue and blue representing normal tissue. (*P < 0.05).

We first analyzed PAQR3 expression levels in pan-cancer cell lines using the CCLE database, and the results showed that PAQR3 expression level varied (Fig. 1a). It is expressed at very high level in NB (neuroblastoma), while very low level in PRAD (prostate adenocarcinoma). Then, we analyzed the differential expression of PAQR3 in pan-cancer in the TCGA database (Fig. 1b), and the data suggested that the expression level of PAQR3 in CHOL, ESCA, HNSC, LIHC, LUAD and LUSC tumors was significantly higher than that in normal tissues. However, the expression level of PAQR3 in BRCA, KIRC, KIRP, PRAD, READ, THCA and UCEC tumors was lower than that in normal tissues. Furthermore, the differential expression of PAQR3 in tumor tissues and normal tissues was analyzed by GEPIA2 (Fig. 1c). The results indicated that PAQR3 was highly expressed in CHOL, DLBC, PAAD, PCPG, and THYM tumors, while the expression level in TGCT was lower than that in normal tissues. This indicates that PAQR3 affects the occurrence and development of tumors through different pathways in different types of tumor tissues.

### PAQR3 was associated with prognosis by pan-cancer analysis

**Figure 2:**
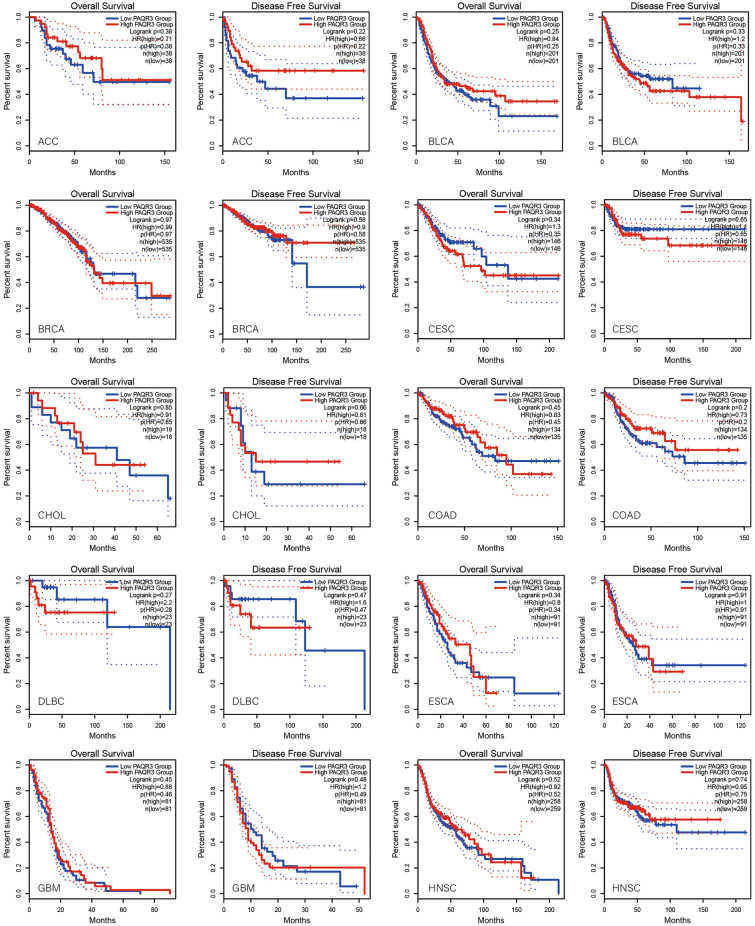

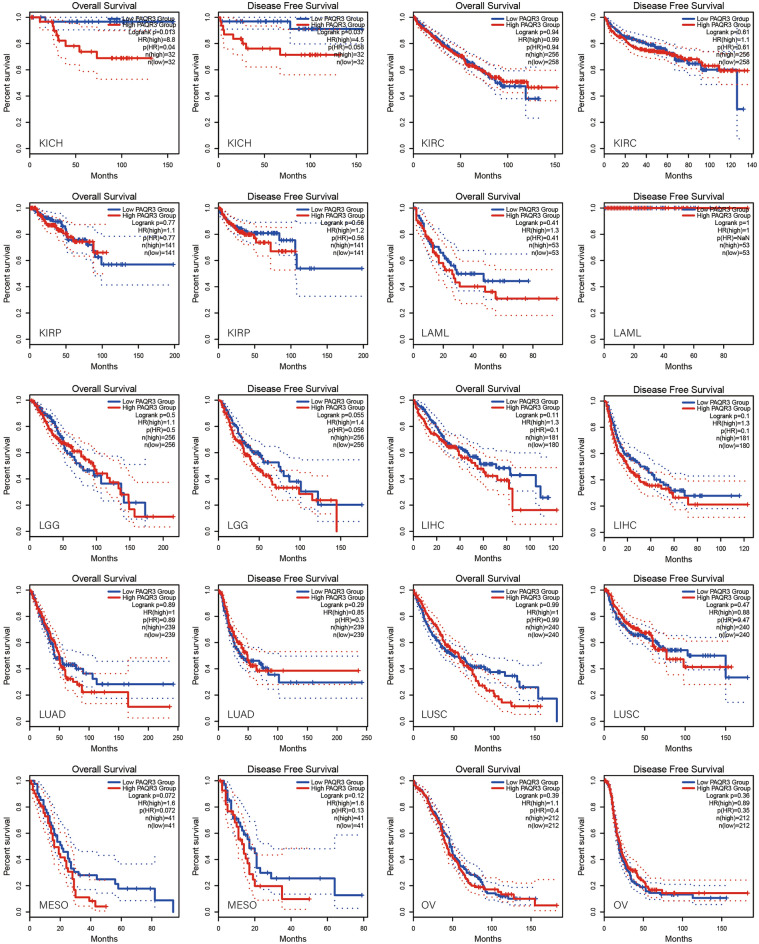

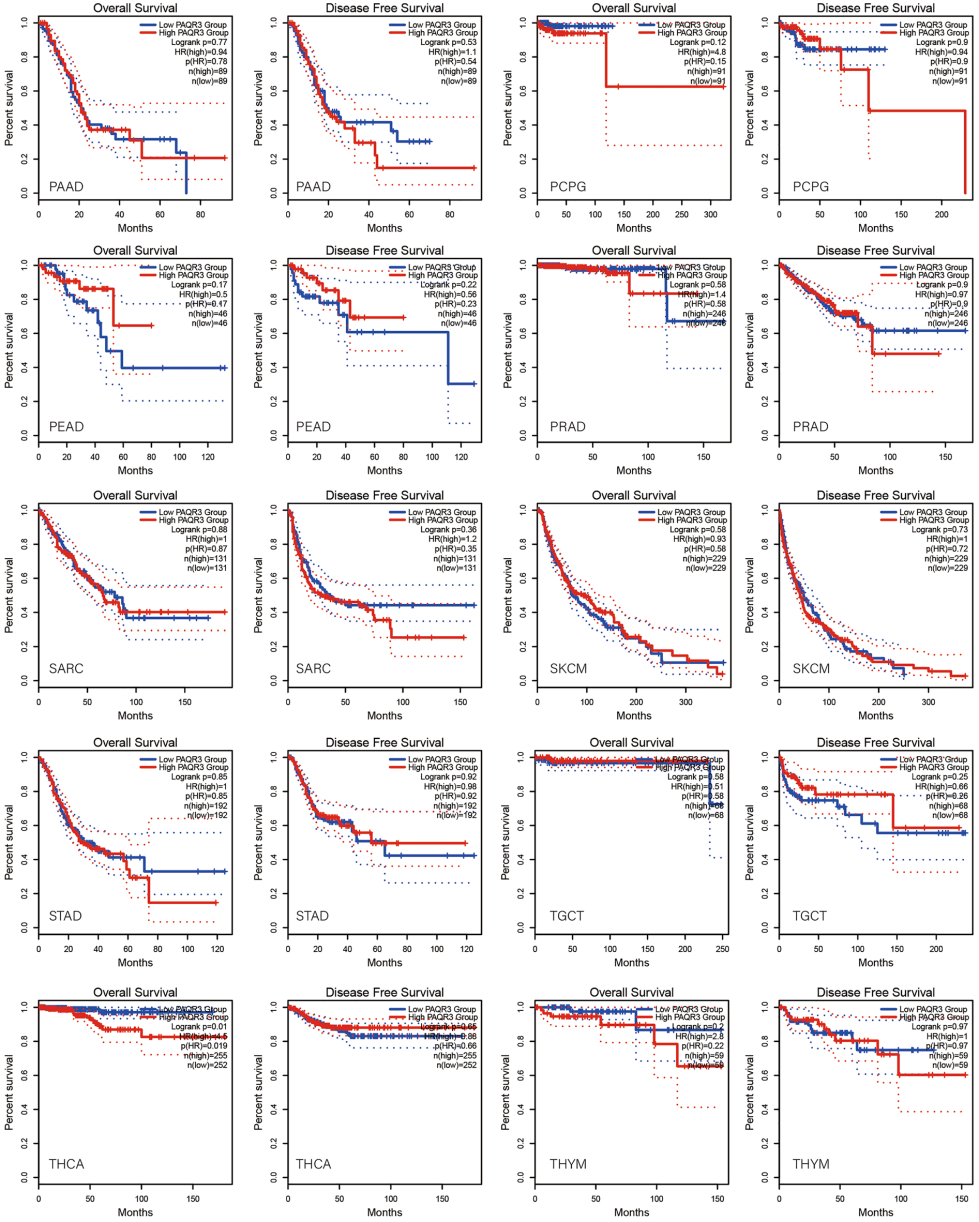

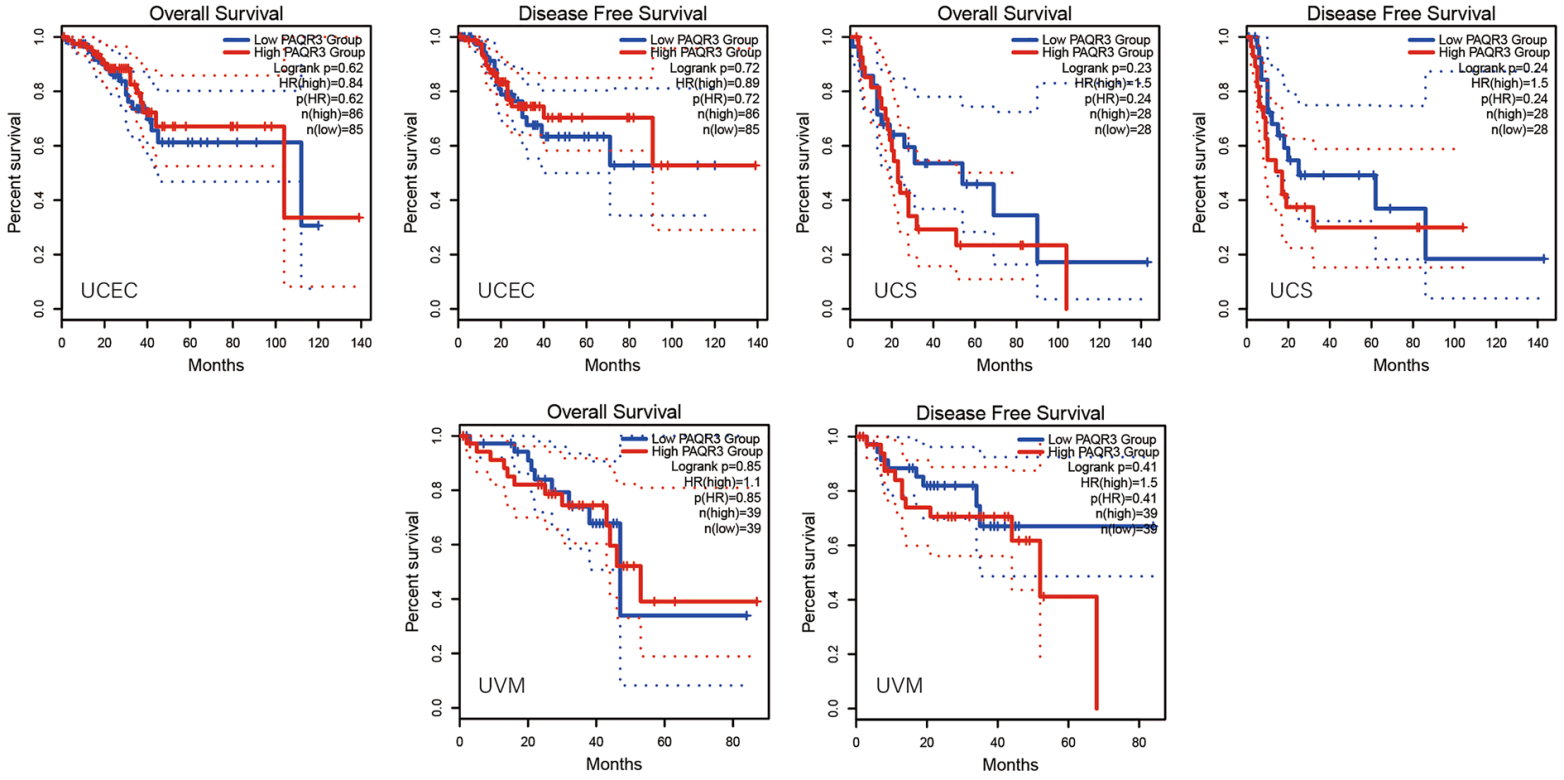
Kaplan–Meier survival curve analysis of the correlation between high and low PAQR3 expression groups in GEPIA 2 database and human tumor OS and DFS. The group with high PAQR3 expression in KICH had worse OS and DFS.

**Figure 3:**
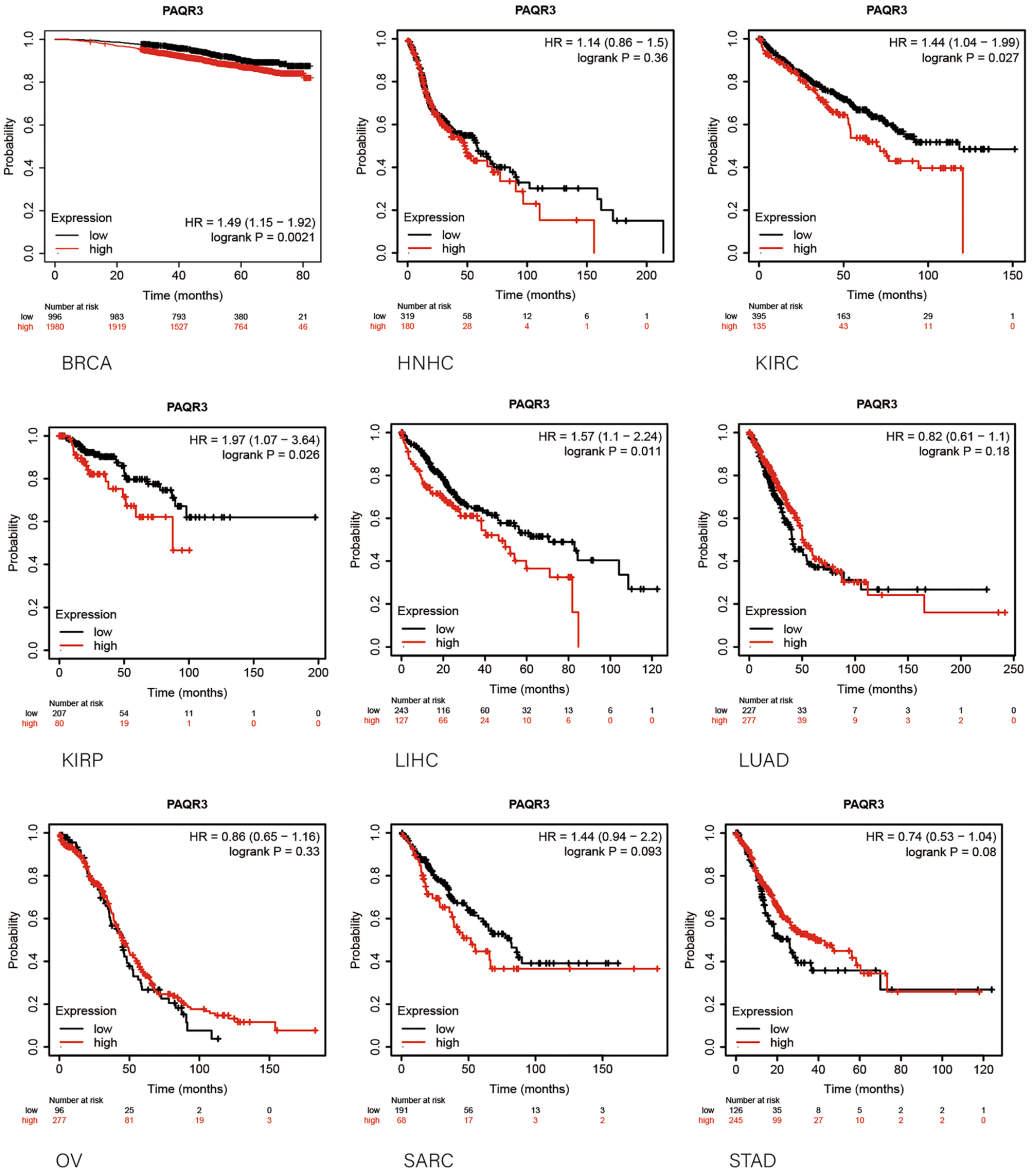
Survival analysis of PAQR3 expression level in tumors was conducted in Kaplan–Meier plotter database. The group with high PAQR3 expression in BRCA, KIRC, KIRP and LIHC had worse OS.

GEPIA2 database was used to analyze the correlation between PAQR3 and OS and DFS in pan-cancer (Fig. 2). The data showed that the expression level of PAQR3, as a cancer promoting factor, was correlated with the OS of THCA. The higher the expression level of PAQR3, the worse the OS of THCA patients. Kaplan–Meier plotter database was then used to analyze the correlation between PAQR3 and prognosis (Fig. 3). The results also showed that patients with high PAQR3 expression in BRCA, KIRC, KIRP, and LIHC tumors had worse OS, indicating that PAQR3 was a risk factor affecting prognosis in these tumor types. PAQR3 expression did not significantly affect OS and DFS in the remaining tumor types.

**Figure 4:**
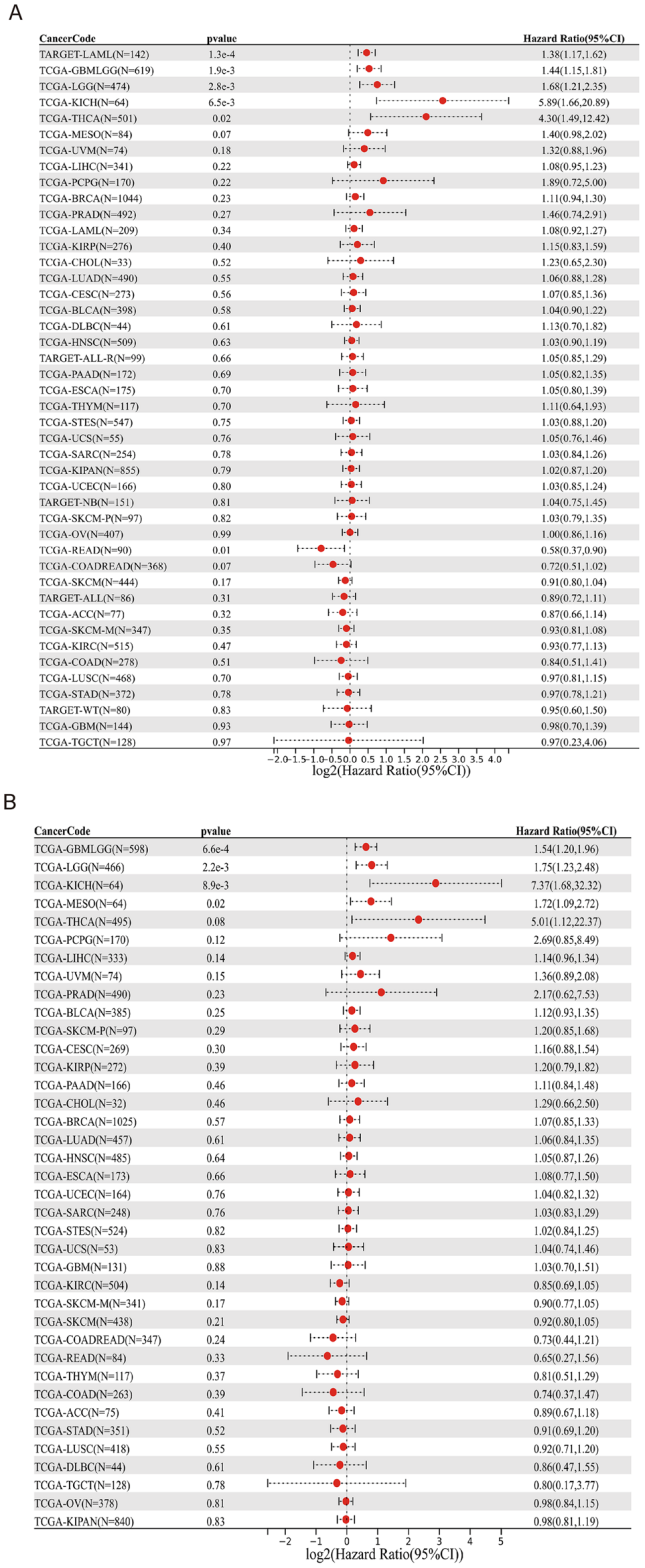

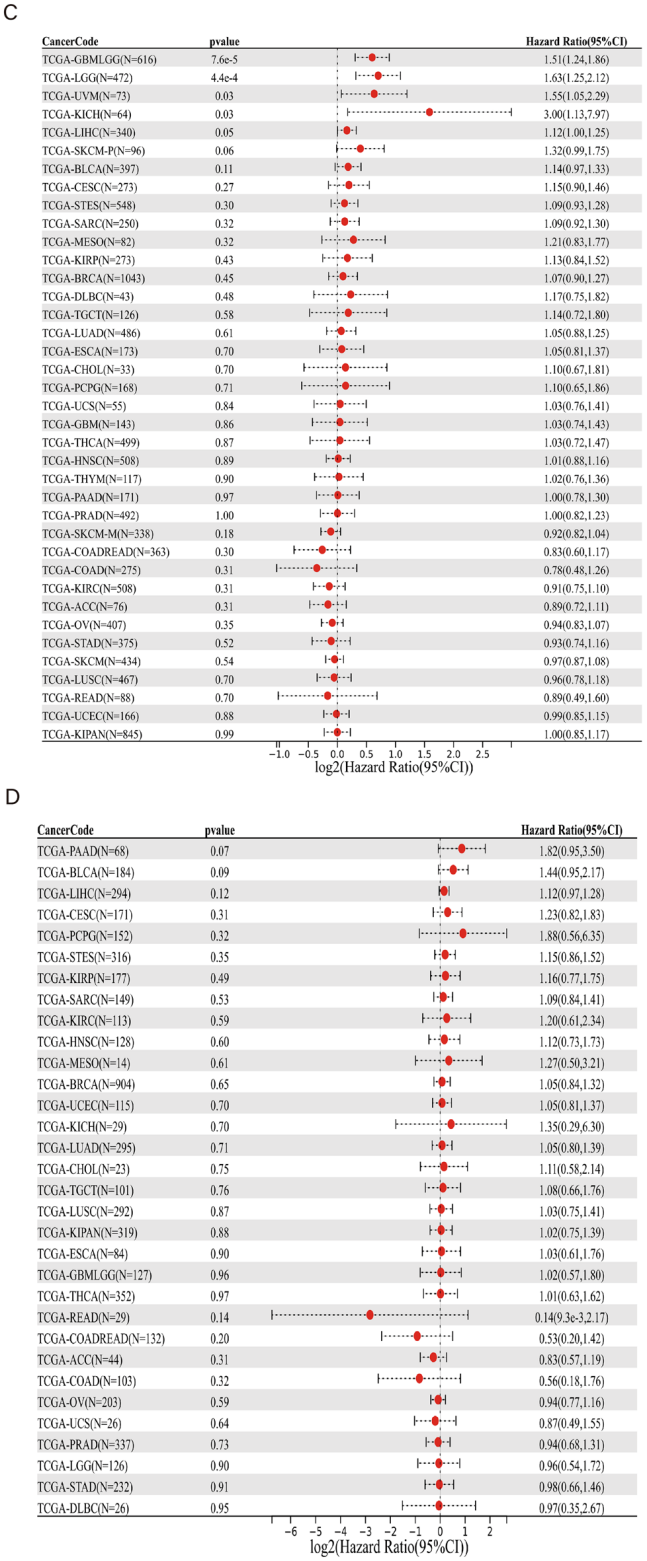
Correlation between PAQR3 expression level and OS (**A**), DSS (**B**), DFI (**C**), PFI (**D**). The group with high PAQR3 expression in TARGET-LAML, TCGA-GBMLGG, TCGA-LGG, TCGA-KICH, TCGA-THCA had positive relation with OS, DSS, and the relation is negative between TCGA-READ PAQR3 expression and OS. The group with high PAQR3 expression in TCGA-GBMLGG, TCGA-LGG, TCGA-UVM, TCGA-KICH, had positive relation with DFI.

Then, we used Sanger Box tool to analyze the correlation between PAQR3 expression level, OS, DSS, PFI and DFI (Fig. 4). After analyzing the data of 32 kinds of tumors, we found that high PAQR3 expression in GBMLGG, LGG, KICH, MESO and THCA was a risk factor for worse DSS (Fig. 4b). The PFI was shorter in the GBMLGG, LGG, UVM, KICH group with high PAQR3 expression (Fig. 4c), the OS was shorter in LAML, GBMLGG, LGG, KICH, THCA group with high PAQR3 expression (Fig. 4a), and the OS was longer in READ group with high PAQR3 expression. In addition, the expression level of PAQR3 did not show significant correlation in the DFI analysis.

### PAQR3 genomic alteration is present in a variety of tumor types

**Figure 5:**
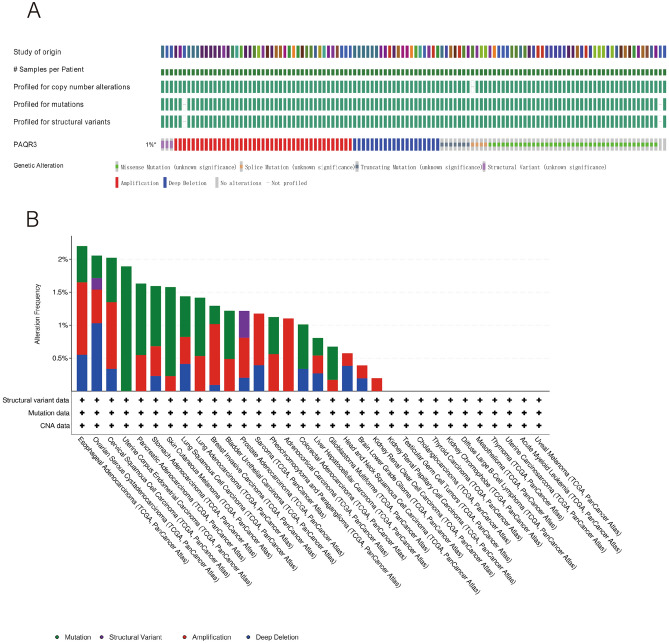

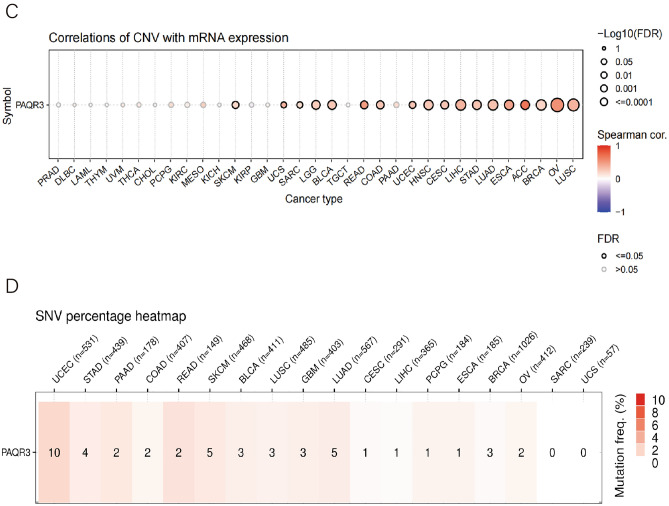
Analysis of PAQR3 genomic alteration in pan-cancer using cBioPortal database. Mutations in the PAQR3 genome were present in 1% of patients (**A**,**B**). The most common type of mutation in PAQR3 is the missense mutation, in addition, common mutation types include amplification, deep deletion, splice mutation, truncating mutation, structural variant. In the tumor samples with PAQR3 alteration, mutation was found in all except PRAD and ACC (adrenocortical carcinoma), HNSC, BLGG (brain low-grade glioma) and KIRC. The frequency of mutation in UCEC was the highest, while the total alteration frequency of EAC was the highest. The correlation between PAQR3 expression and CNV in different tumors was analyzed by GSCA (**C**). The SNV frequency of PAQR3 in different tumors was analyzed by GSCA (**D**). PAQR3 was closely associated with CNV in 18 tumors, especially in OV, LUSC, and ACC. The SNV frequency analyzed by GSCA database showed that PAQR3 has a high SNV frequency in UCEC, SKCM, LUAD, and the highest frequency in UCEC.

The PAQR3 genomic alteration in pan-cancer was analyzed by cBioPortal database (Fig. 5), and it was found that 1% of patients had PAQR3 genomic mutation, Amplification was the most dominant type of mutation in PAQR3, followed by Deep Deletion and Missense Mutation (Fig. 5a). In the tumors analyzed, PAQR3 had the highest proportion of genomic alteration in EAC (esophageal adenocarcinoma), and the type was mainly amplification. High PAQR3 deep deletion in OV (ovarian serous cystadenocarcinoma), both mutation and amplification are common in CESC (cervical squamous cell carcinoma), where amplification is more frequent. UCEC (uterine corpus endometrial carcinoma) had the highest mutation alteration frequency. (Fig. 5b).

Then, we further analyzed the correlation between PAQR3 expression level, CNV (copy number variation) and SNV (single nucleotide variation) using GSCA database (Fig. 5c,d). CNV is a structural mutation that duplicates part of the genome structure, affecting the replication or deletion of a significant number of base pairs. Such copy number mutations vary among individuals and play a key role in disease phenotypic variation^19^. SNV is a general term for a single nucleotide change in a DNA sequence. It is often used in molecular diagnosis. Analysis showed that PAQR3 was closely associated with CNV in 18 tumors, especially in OV, LUSC and ACC (Fig. 5c). The SNV frequency of PAQR3 analyzed by GSCA database showed that the SNV frequency of PAQR3 was high in UCEC, SKCM, LUAD, and the highest in UCEC (Fig. 5d). These data suggest that PAQR3 has genomic mutation in various types of tumor tissues and may be involved in the occurrence and development of tumors.

### PAQR3 gene promoter methylation levels were analyzed by pan-cancer

**Figure 6:**
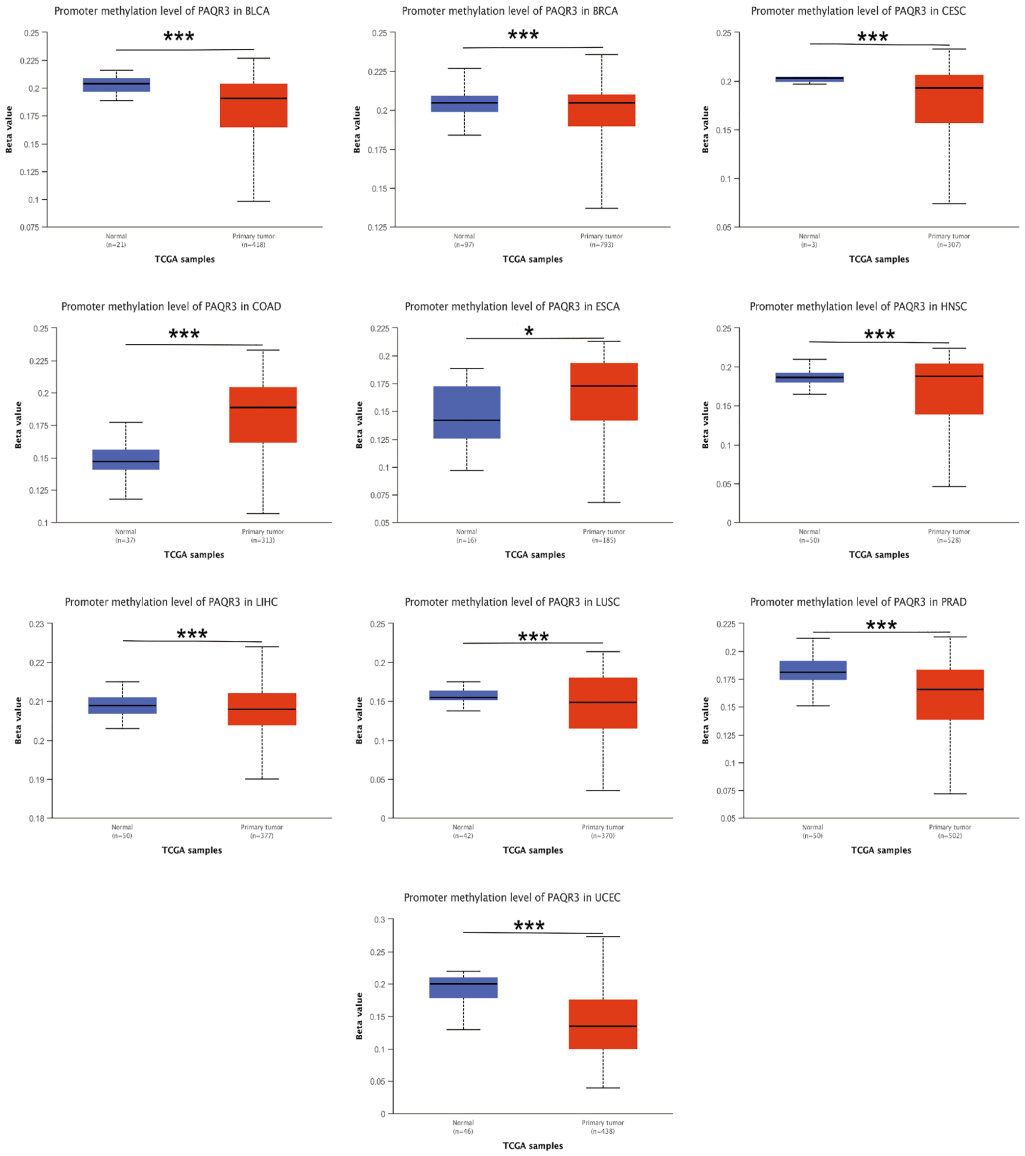
PAQR3 promoter methylation levels in different tumors and normal tissues were analyzed by UALCAN. The PAQR3 promoter methylation level in tumor tissues was significantly changed compared with that in normal tissues in 10 types of tumors. Among them, the PAQR3 promoter methylation level in tumor tissues was significantly higher than that in normal tissues in COAD and ESCA, while the result was opposite in BLCA, PRAD and UCEC, and the PAQR3 promoter methylation level in tumor tissues was significantly lower than that in normal tissues.

Changes in DNA methylation are an important component of cancer development. In tumor cells, abnormal hypermethylation of the gene promoter CpG island leads to transcriptional silencing, a phenotype that can still be inherited by daughter cells after cell division^20^. Hypomethylation is associated with chromosomal instability and imprinting loss, while hypermethylation is associated with promoters that can be secondary to gene silencing and have potential as epigenetic therapy targets^21^. UALCAN database was used to analyze the methylation level of PAQR3 gene promoter, and the analysis showed that in 10 types of tumors, PAQR3 promoter methylation level in tumor tissues showed significant changes compared with normal tissues (Fig. 6). The methylation level of PAQR3 promoter in tumor tissues was significantly higher than that in normal tissues in COAD and ESCA, while the opposite was true in BLCA, PRAD and UCEC, and the methylation level of PAQR3 promoter in tumor tissues was significantly lower than that in normal tissues. These partial results suggest that the regulation of PAQR3 promoter methylation is distinguishing in different tumors.

### PAQR3 expression level was associated with TME immune subtypes

The study of TME (tumor microenvironment) provides new opportunities to deconstruct tumor complexity. In a variety of cancers, TME subtypes as immunotherapy markers are associated with patient response to immunotherapy. Comprehensive analysis of TME subtypes can help identify new biomarkers and personalize treatment^22^. According to the five immuno-characteristics of macrophage/monocyte, lymphocyte infiltration, TGF-β response, IFN-γ response and wound healing, six immune subtypes C1–C6 were derived from the cluster analysis of non-hematologic tumors. The expression of angiogenic genes in C1 (wound healing) subtype was up-regulated, and Th2 cells tended to be immune-infiltrated. In C2 (IFN-γ Dominant) subtype, macrophages had the highest M1/M2 polarization and the most abundant TCR diversity. C3 (inflammation) The Th17 and Th1 genes of this subtype are upregulated, and the proliferation rate of tumor cells is low or moderate. Th1 of C4 (lymphocyte depletion) is inhibited, while M2 is highly reactive. This subtype of C5 (Immunologically Quiet) is characterized by the lowest lymphocyte response and the highest macrophage response, mainly M2 macrophages. C6 (TGF-β Dominant) subgroup was the one with the highest TGF-β signal and high lymphatic infiltration^23^.

**Figure 7:**
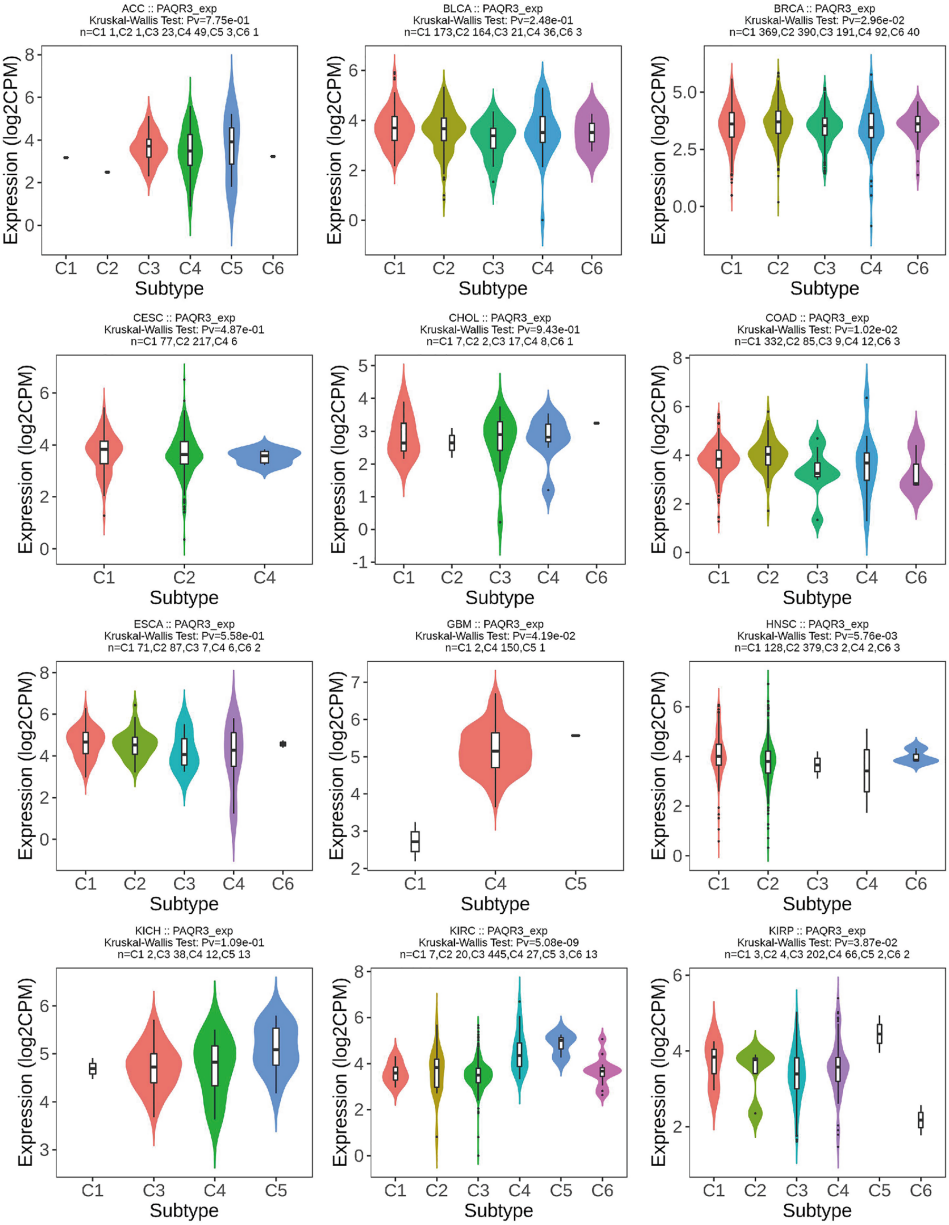

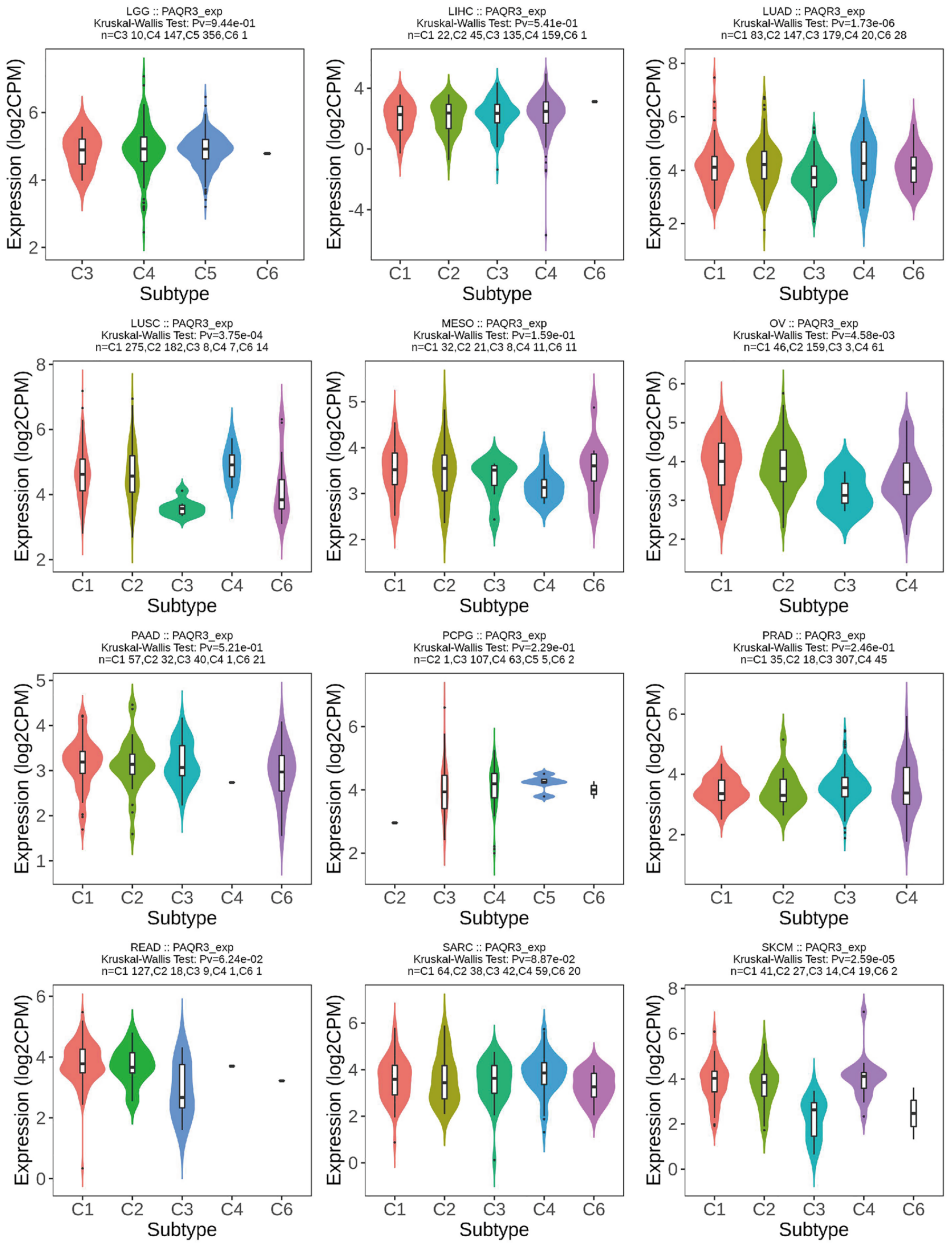

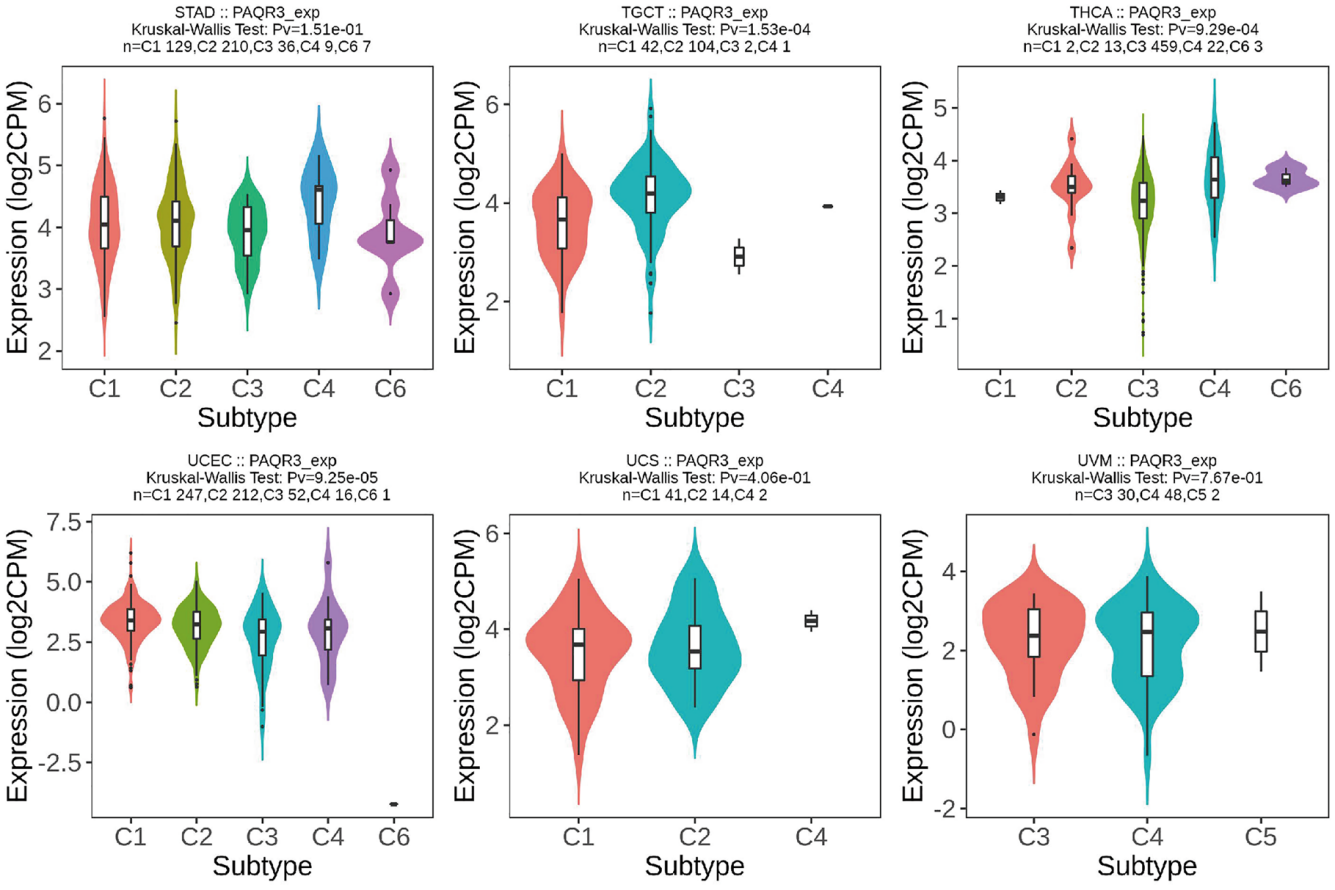
Correlation between PAQR3 expression level and immune subtypes in pan-cancer, including six subtypes C1-C6. The expression level of PAQR3 is different in different immune subtypes of the same tumor. For example, the expression level of PAQR3 in C3 subtype was significantly lower than that of other subtypes in COAD, LUSC, OV and SKCM, and the expression level of PAQR3 in MESO C4 subtype was significantly lower than that of other subtypes. However, the expression level of PAQR3 in C4 in GBM and KIRC was significantly increased. This suggests that the expression level of PAQR3 is related to immune typing.

The TISIDB immunological website found that PAQR3 expression level was diverse in different immune subtypes of the same tumor (Fig. 7). For example, PAQR3 expression level of C3 subtype were significantly lower than those of other subtypes in COAD, LUSC, OV and SKCM, and the expression level of PAQR3 in MESO C4 subtype was significantly lower than that of other subtypes. However, the expression level of PAQR3 of C4 was significantly increased in GBM and KIRC. This suggests that the expression level of PAQR3 is related to immune typing.

### The relevance of PAQR3 expression level to TMB, MSI, NEO, purity

A tumor is a collection of cancer cells, host cells, secretory factors, and extracellular matrix. Non-cancerous components make up the tumor microenvironment (TME), a complex and constantly changing entity, and current evidence suggests that TME is an active participant in cancer progression^24–30^. TMB, MSI, NEO, Purity are important biomarkers to measure the tumor microenvironment^26^.

Tumor mutation burden (TMB) is to study the number of non-heritable mutations per million bases in the gene sequence, which is one of the genetic characteristics of tumor tissue^24^. Associations between TMB levels and ICI efficacy have been reported in various tumors, and TMB has shown potential as a predictive biomarker^25^. Microsatellite instability (MSI) is a genetic hypermutation caused by damaged DNA mismatch repair (MMR) and can be used as an indicator to determine the prognosis of cancer treatment^26^. Neoantigen (NEO) includes antigen produced by tumor viruses integrated into the genome and antigen produced by mutant proteins. Neoantigen (NEO) is highly immunogenic and tumor heterogeneous, and therapies targeting NEO are currently being developed. Fortunately, a number of clinical trials have demonstrated the safety and efficacy of the vaccine^27,28^. Purity represents the proportion of cancer cells in the tumor tissue. Non-tumor cells in solid tumors include stromal cells and immune cells, which are important components of TME and play an important role in tumor progression, drug resistance and prognosis evaluation^29^.

**Figure 8:**
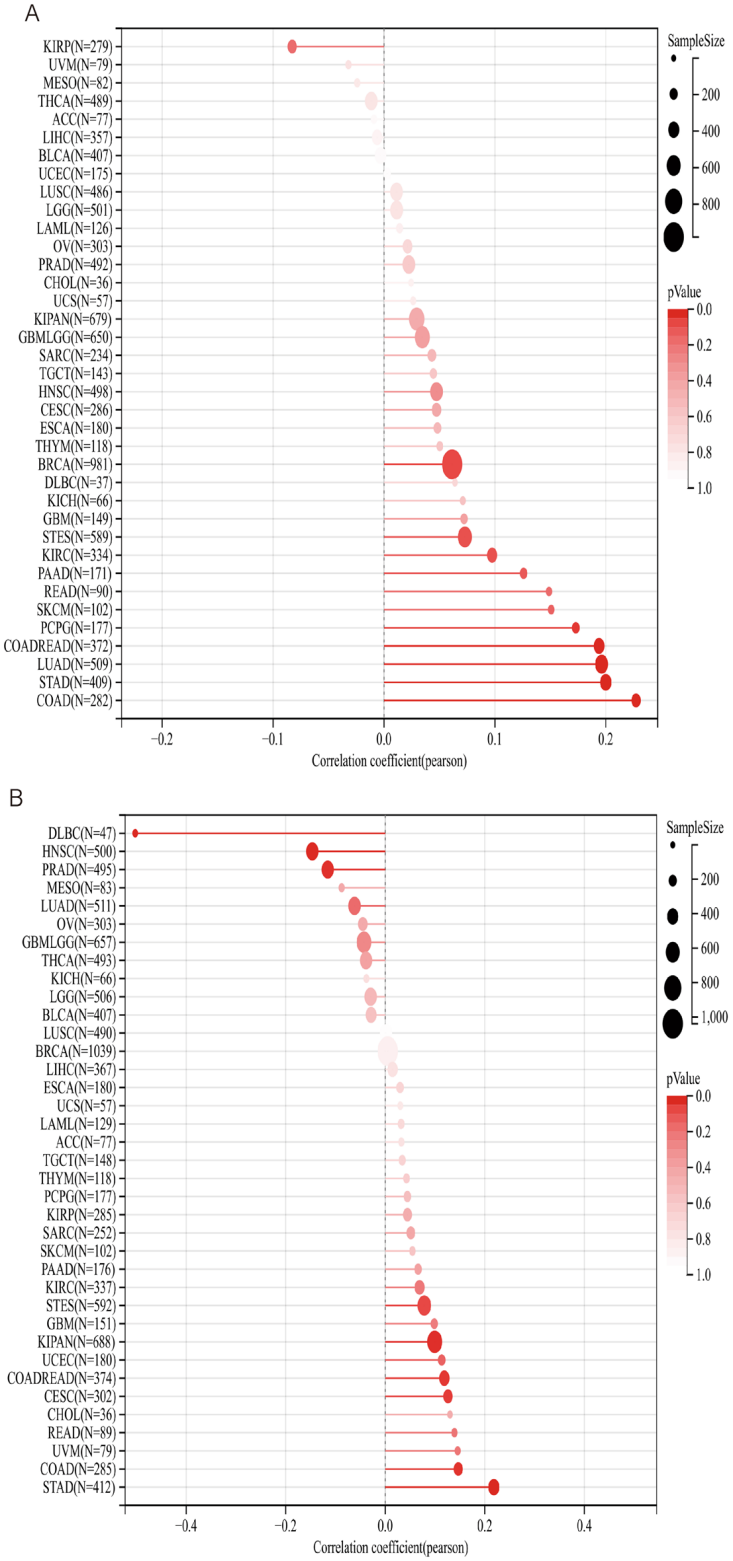

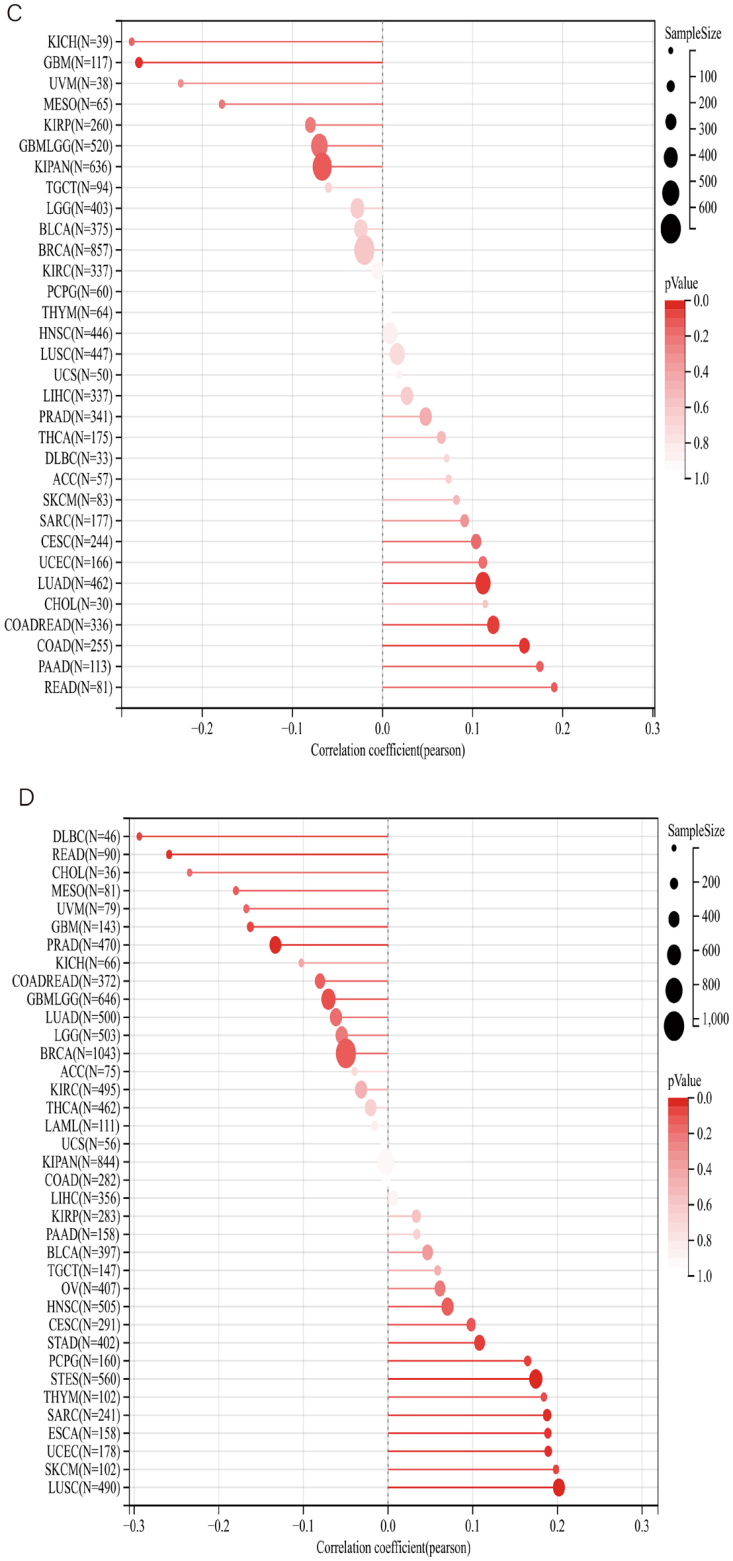
Correlation between PAQR3 expression level and TMB (**A**), MSI (**B**), NEO (**C**), and Purity (**D**). In the TMB index, the expression level of PAQR3 was significantly positively correlated with 11 types of tumors (BRCA, STES, KIRC, PAAD, READ, SKCM, PCPG, COADREAD, LUAD, STAD), and significantly negatively correlated with KIRP. The expression level of PAQR3 in MSI was significantly correlated with the MSI of GBM, KIPAN, UCEC, COADREAD, COAD and STAD, respectively, and significantly negatively correlated with DLBC, HNSC and PRAD. PAQR3 was significantly correlated with NEO, and analysis showed that it was positively correlated with 7 types of tumors (CESC, UCEC, LUAD, COADREAD, READ, PAAD) and negatively correlated with another 7 types of tumors (KICH, GBM, UVM, MESO, KIRP, GBMLGG, KIPAN). In addition, the analysis found that PAQR3 expression level was significantly correlated with Purity of 25 types of tumors, among which it showed a negative correlation in 12 types of tumors (DLBC, READ, CHOL, MESO, UVM, GBM, PRAD, COADREAD, GBMLGG, LUAD, LGG, BRCA). There was a significant positive correlation with another 13 types of tumors (BLCA, OV, HNSC, CESC, STAD, PCPG, STES, THYM, SARC, ESCA, UCEC, SKCM, LUSC).

Pearson correlation coefficient was used to analyze the PAQR3 expression level and the correlation between TMB, MSI, NEO and Purity (Fig. 8). The analysis showed that the expression level of PAQR3 in the TMB index was significantly positively correlated with 11 types of tumors (BRCA, STES, KIRC, PAAD, READ, SKCM, PCPG, COADREAD, LUAD, STAD), and negatively correlated with KIRP (Fig. 8a). The expression level of PAQR3 in MSI was significantly correlated with the MSI of GBM, KIPAN, UCEC, COADREAD, COAD and STAD, respectively, and significantly negatively correlated with DLBC, HNSC and PRAD (Fig. 8b). PAQR3 was significantly associated with NEO. Analysis showed that PaQR3 was positively associated with 7 tumors (CESC, UCEC, LUAD, COADREAD, READ, PAAD) and negatively associated with another 7 tumors (KICH, GBM, UVM, MESO, KIRP, GBMLGG, KIPAN) (Fig. 8c). In addition, the analysis found that there was a significant correlation between the expression level of PAQR3 and Purity in 25 types of tumors, among which it showed a negative correlation in 12 types of tumors (DLBC, READ, CHOL, MESO, UVM, GBM, PRAD, COADREAD, GBMLGG, LUAD, LGG, BRCA). There was a significant positive correlation with the other 13 types of tumors (BLCA, OV, HNSC, CESC, STAD, PCPG, STES, THYM, SARC, ESCA, UCEC, SKCM, LUSC) (Fig. 8d). These results indicate that PAQR3 expression level is related to biomarkers in a variety of tumor microenvironments in various tumors, and therefore has the potential to be a potential target for tumor immunotherapy.

### The relevance of PAQR3 expression level to stromal score, immune score

**Figure 9:**
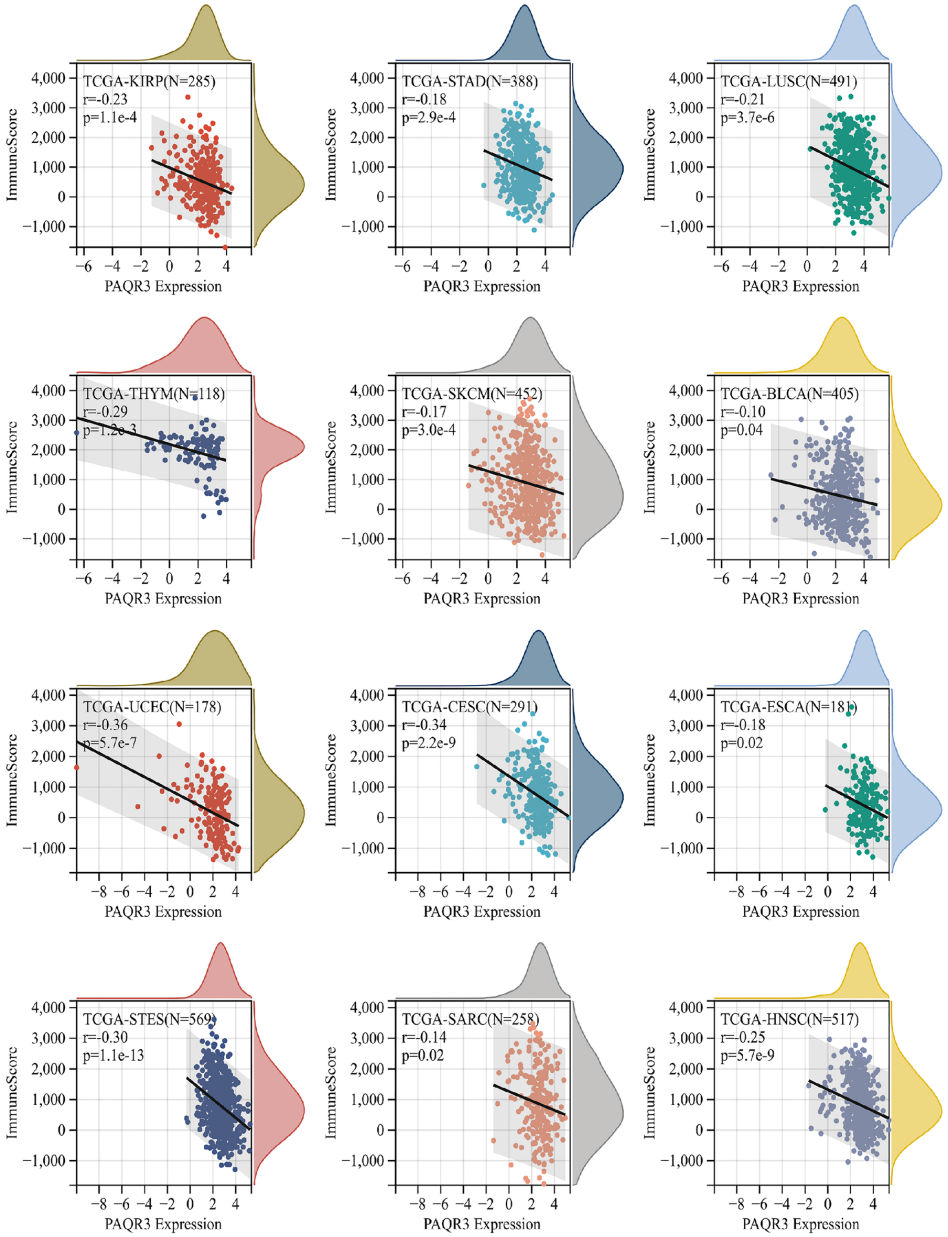

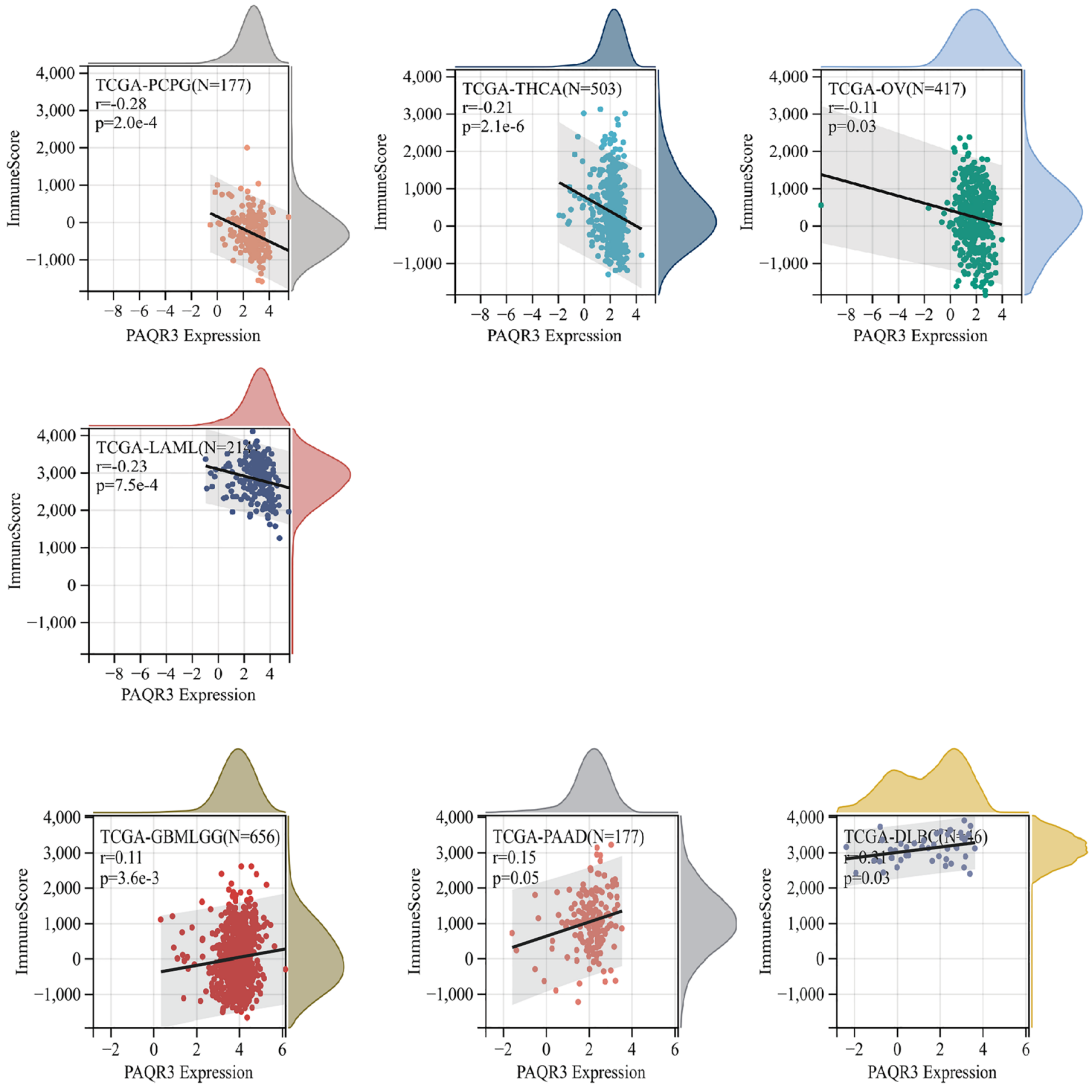
Correlation between PAQR3 expression level and immune score. In terms of Immune Score, PAQR3 expression levels were associated with 19 types of tumors, including 3 positive correlations (GBMLGG, PAAD, DLBCL) and 16 negative correlations.

**Figure 10:**
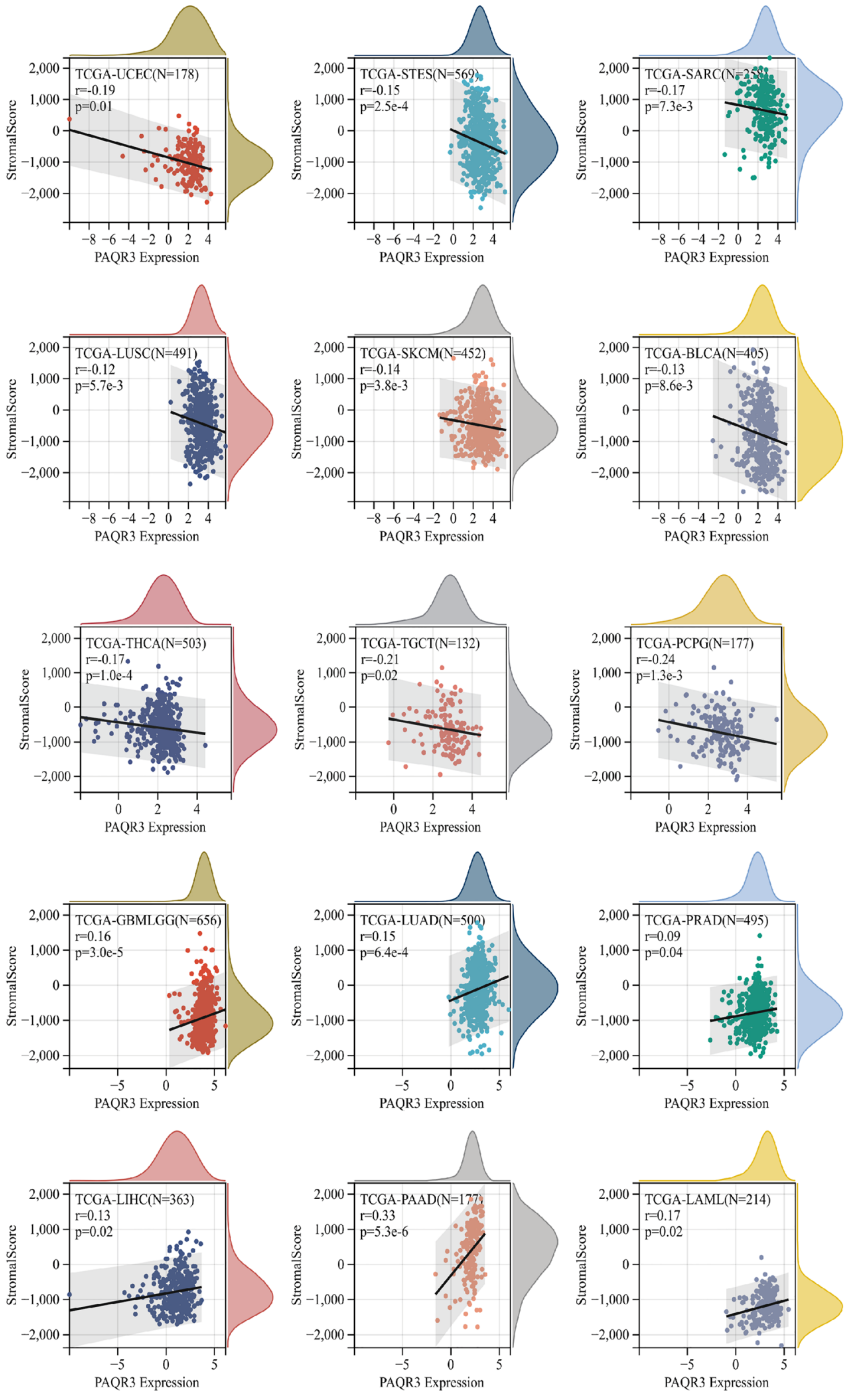
Correlation between PAQR3 expression level and stromal score. The expression level of PAQR3 was positively correlated with Stromal Score in 6 types of tumors (GBMLGG, LUAD, PRAD, LIHC, PAAD, LAML), and 9 types of tumors (UCEC, STES, SARC, LUSC, SKCM, BLCA, THCA, TGCT, etc.). PCPG) is negatively correlated.

The Stromal Score and Immune Score of tumor were analyzed by ESTIMATTE, and the correlation between them and the expression of PAQR3 was studied (Figs. 9, 10). Among 39 types of tumors, 6 types of tumors (GBMLGG, LUAD, PRAD, LIHC, PAAD, LAML) showed a positive correlation between PAQR3 expression level and Stromal Score, while 9 types of tumors (UCEC, STES, SARC, LUSC, SKCM, BLCA, THCA, TGCT, PCPG) were negatively correlated (Fig. 10). In terms of Immune Score, PAQR3 expression levels were correlated with 19 types of tumors, including 3 positive correlations (GBMLGG, PAAD, DLBCL) and 16 negative correlations (Fig. 9). This part of the analysis data also proved that PAQR3 is associated with the tumor microenvironment.

### PAQR3 expression level and drug sensitivity

**Figure 11:**
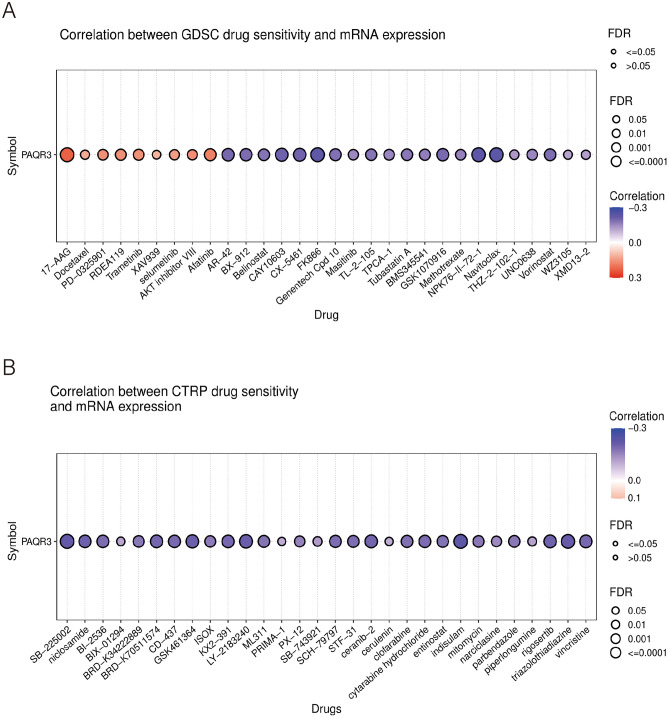
Correlation between PAQR3 mRNA expression level and drug sensitivity. GDSC database (**A**) and CTRP database (**B**). GDSC showed that the expression level of PAQR3 was significantly positively correlated with 17-AAG, PD-0325901, RDEAA119, Trametinib, Selumetinib, AKT inhibitor VII, Afatinib, and conversely with CAY10603, CX-5461, FK866, NPK76-II-72-1 and Navitoclax were negatively correlated. The CTRP database analysis showed that the expression level of PAQR3 was negative correlated with drug sensitivity in 30 drugs, Including niclosamide, clofarabine, cytarabine hydrochloride, indisulam, rigosertib, triazolothiadiazine.

**Figure 12:**
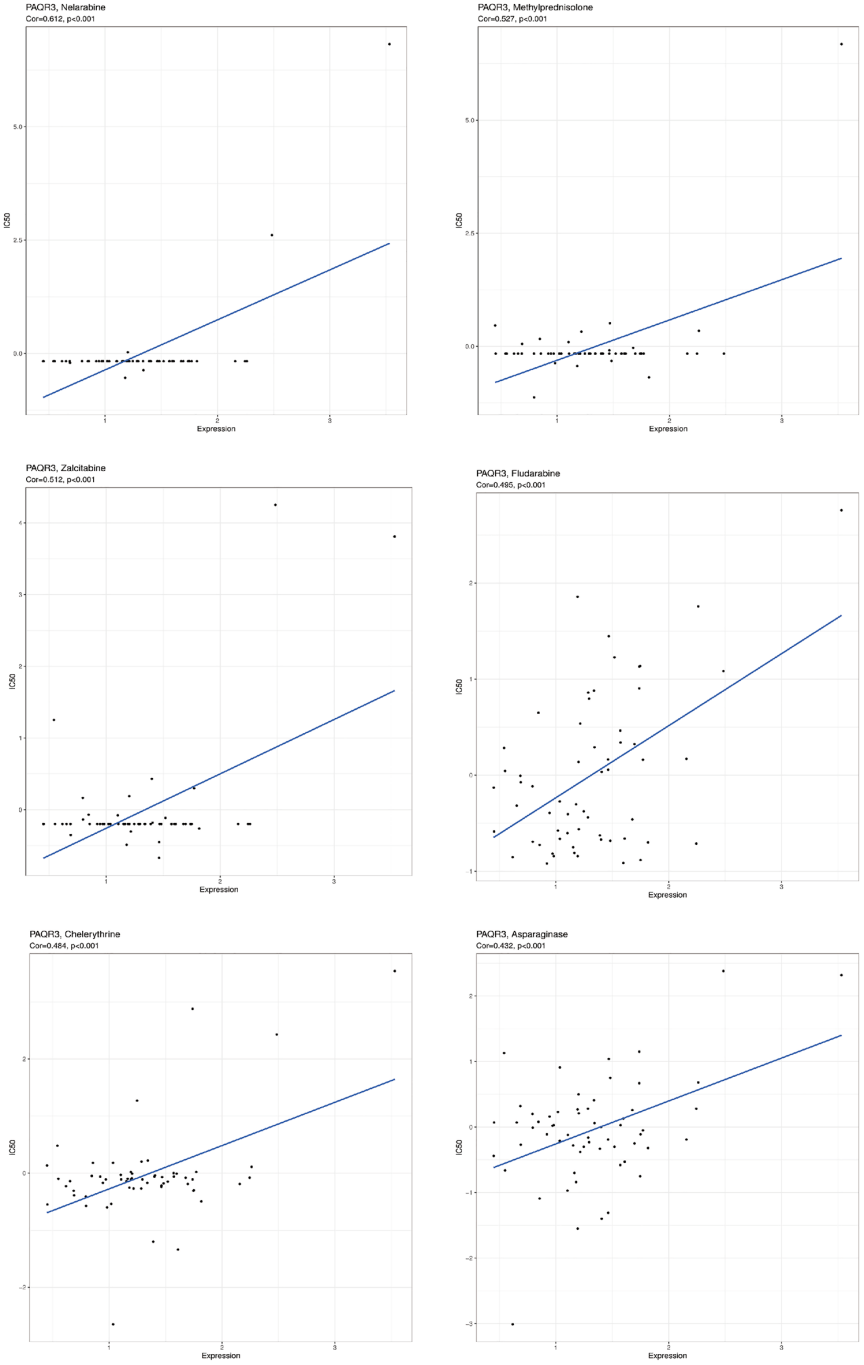
Correlation between PAQR3 expression level and IC50. Six drugs most significantly correlated with PAQR3 expression levels.

Despite the growing diversity of treatments, the metastasis and recurrence of cancer cells and the drug resistance to chemotherapy and radiotherapy caused by their own heterogeneity make the traditional treatment methods ineffective for malignant tumors^31^. In the current tumor treatment, chemotherapy is recognized as the most effective treatment. However, it has been reported in many literatures that conventional chemotherapy drugs, due to their non-specific distribution, lead to poor bioavailability and rapid plasma clearance^32,33^. The development of tumor drug resistance is associated with abnormal gene regulation^34^. It is still necessary to understand the specific mechanism of tumor resistance. We then examined the association between PAQR3 and drug sensitivity using GSCA website analysis (Figs. 11, 12). The results of GSCA analysis showed that the expression level of PAQR3 was significantly positively correlated with 17-AAG, PD-0325901, RDEAA119, Trametinib, Selumetinib, AKT inhibitor VIII, Afatinib, and conversely correlated with CAY10603, CX-5461, FK866, NPK76-II-72-1 and Navitoclax (Fig. 11a). The CTRP database analysis showed that PAQR3 expression level was negatively correlated with the sensitivity of 30 drugs including niclosamide, clofarabine, cytarabine hydrochloride, indisulam, rigosertib, triazolothiadiazine (Fig. 11b). Using CellMiner to analyze correlations between drug data and PAQR3 expression levels in 60 tumor cell types with tumor cell lines (Fig. 12), result showed that there was a significant positive correlation between the expression level of PAQR3 with 6 classes of drugs, these drugs include Asparaginase, Chelerythrine, Fludarabine, Methylprednisolone, Nelarabine, Zalcitabine. These results suggest that the occurrence and development of drug resistance in tumors may be related to the abnormal expression of PAQR3, and provide potential candidate targets for accurate treatment of tumors.

## Discussion

Cancer is not easy to detect in the early stage, and the characteristics of metastases in the middle and late stages and resistance to treatment due to its own heterogeneity make cancer a major cause of death worldwide^35,36^. Chemotherapy is still the first-line treatment for most tumors at present, but the poor efficacy and side effects revealed in clinical application have seriously affected the course of tumor treatment and patients' life^37^. The ultimate goal of cancer drugs is to selectively kill tumor cells without damaging surrounding healthy cells and tissues, a goal that has yet to be achieved. Currently, targeted therapy is in full swing through the development of antibodies and small molecule inhibitors by targeting molecules^38^. Although more and more mutations and targetable molecules have been discovered, the efficacy of targeted therapy remains unsatisfactory, with many patients becoming insensitive to small molecule inhibitors. Studies on the correlation between tumor and immunity have also provided new insights into tumor therapy^39^, from which the development of PD-L1 inhibitors can significantly prolong the survival of patients^40^. However, the heterogeneity of tumor itself and the relationship between tumor and tumor microenvironment have not been systematically and comprehensively interpreted, and current therapeutic approaches are still imperfect. Therefore, it is urgent to continue to explore new targets for precision therapy^41^.

In order to provide valuable targets for tumor precision therapy, we used bioinformation technology to analyze differences in gene expression at the transcriptome level in pan-cancer. We found widespread and significant changes in PAQR3 expression level in the TCGA and GTEx databases in pan-cancer. Studies of this molecule PAQR3 in specific tumor types have shown that PAQR3 is involved in the generation of tumor drug resistance and the regulation of cell death. Its role in NSCLC, BRCA and PA has been demonstrated through experiments. Further, we found that PAQR3 had prognostic value, and its expression level was closely correlated with prognostic indicators OS, DFS, DSS, DFI and PFI. High PAQR3 expression in a variety of tumors is associated with poorer prognosis, suggesting that PAQR3 has the potential to be used as a prognostic marker for multiple types of tumors. Secondly, by analyzing PAQR3 from the perspective of epigenetics, we found that the methylation level of PAQR3 gene promoter was significantly changed in 10 types of tumors, and this epigenetic alteration led to the change of PAQR3 expression level in these tumors and could be used as a corresponding tumor marker^42^.

The tumor microenvironment (TME) is a complex and evolving entity, consisting primarily of immune cells, stromal cells, blood vessels, and extracellular matrix^43–45^. In the process of tumor growth, cancer cells and TME components form a dynamic correlation, promoting cancer cell survival, invasion and metastasis. Currently, more and more studies have reported the target of TME intervention in tumor^45–47^. The classification of immune subtypes enables researchers to more intuitively understand the immune status of the tumor environment^23^. We found that the expression level of PAQR3 was significantly different in different immune subtypes, suggesting that PAQR3 may be involved in the immune regulation of tumor microenvironment and promote the differentiation of TME to different immune subtypes. TMB, MSI and NEO are important indicators to evaluate the tumor microenvironment. TMB is defined as the number of non-inherited mutations per million bases in the gene sequence studied, which is one of the genetic characteristics of tumor tissues^4^ and can be used as an indicator to measure the efficacy of immune checkpoint inhibitors (ICI)^43^. Continuously high TMB represents better ICI efficacy. Microsatellite instability (MSI) is a genetic hypermutation caused by damaged DNA mismatch repair (MMR) and can be used as an indicator to determine the prognosis of cancer treatment^26^. Neoantigen (NEO), which was only widely expressed in tumor cells and had strong immunogenic and tumor heterogeneity, is an ideal target for tumor immunotherapy, and vaccines targeting NEO have been tested in clinical trials in a variety of tumors^44^. In this study, we found that the expression level of PAQR3 was significantly related to TMB, MSI and NEO in more than 10 kinds of tumors, and was closely related to tumor purity in 25 kinds of tumors. In addition, PAQR3 expression level was associated with the Immune score and Stromal Score of more than half of the tumor classes. These data suggest that PAQR3 is closely related to TME regulation in a variety of tumors and has potential as a target.

Previous studies have shown that PAQR3 is involved in the resistance of NSCLC to the EGFR inhibitor erlotinib^3^, and in our study, it was found that the expression level of PAQR3 is related to the sensitivity of multiple drugs. Next, we will further determine the mechanism of PAQR3 in drug resistance in different tumors and provide ideas for personalized treatment.

Although this study was cross-analyzed and verified by multiple databases, there are still many deficiencies. The function of PAQR3 in tumors still needs to be further verified by cell and animal experiments. Secondly, although we found that PAQR3 is associated with the regulation of tumor microenvironment, the specific regulatory mechanism remains unclear. Therefore, we will study the function of PAQR3 in tumors and try to develop new anti-cancer drugs targeting PAQR3 to promote the development of precision therapy for tumors in the next.

## Conclusion

Using multi-database transcriptome data to analyze the difference in PAQR3 expression between tumor tissues and normal tissues, we found that PAQR3 was significantly overexpressed in CHOL, ESCA, HNSC, LIHC, LUAD and LUSC. However, the expression levels of PAQR3 RNA in HNSC, LIHC, LUAD and LUSC tumor tissues are very different, which may indicate that the current classification criteria cannot accurately separate the subgroups with low expression of PAQR3 from those with high expression of PAQR3 in tumor groups. In the future, we still need to find a classification standard that can achieve this purpose. Thus, the relationship between PAQR3 expression level and patient prognosis and treatment can be more accurately reflected. Our studies also found that there are relatively high PAQR3 alteration frequency in ESCA, OV, CESC and UCEC, and the expression level of PAQR3 alteration in OV and ESCA is closely related to CNV, and the Frequency of SNV alteration in UCEC is very high. Therefore, we believe that OV and UCEC are highly concerned about the expression and mutation of PAQR3, and STAD, LUAD and SKCM also have high frequency SNV and are closely related to CNV, so further study may provide some new thinking for this molecule as a prognostic or therapeutic target.

In addition, we studied the relationship between PAQR3 and tumor microenvironment and drug IC50, and found that the expression level of PAQR3 was correlated with HSP90 inhibitor Tanespimycin (17-AAG), chemotherapy drugs Docetaxel, Nelarabine, Fludarabine, Zalcitabine (nucleoside analogue reverse transcriptase inhibitor (NRTI)), MEK inhibitor Mirdametinib (PD-0325901), Refametinib, Trametinib, Selumetinib, Wnt/beta-catenin Inhibitor XAV-939 and HER2 inhibitor Afatinib. At present, several studies have mentioned the correlation between PAQR3 and MEK^48–50^, cell cycle^7,51,52^.

These results suggest that PAQR3 may be a potential immunotherapeutic target with important implications for tumor therapy. However, there are still some problems to be solved. For example, it has been mentioned in some studies that PAQR3 can inhibit MEK pathway activity in liver cancer, colorectal cancer and other tumors, and our analysis found that the expression level of PAQR3 is positively correlated with the effect of MEK inhibitors. Clarifying in which tumors and subtypes PAQR3 can be used as an indicator of drug use is an urgent problem for the future.

## Data Availability

Transcriptome data and related clinical data were downloaded from TCGA through the Harmonized Cancer Datasets Genomic Data Commons Data Portal (https://portal.gdc.cancer.gov/). TIMER2.0, GEPIA2, and GSCA. TIMER (http://timer.cistrome.org/) is a webpage based on the TCGA. GEPIA2 (http://gepia2.cancer-pku.cn/#index) combines the TCGA and GTEx databases for differential analysis. GSCA (http://bioinfo.life.hust.edu.cn/GSCA/#/) integrates the drug sensitivity and transcriptomics data of cancer cell lines in GDSC (genomics of drug sensitivity in cancer) and CTRP (the cancer therapeutics’ response portal). cBioPortal (https://www.cbioportal.org) is a platform for exploring multidimensional cancer genomic data based on the TCGA. UALCAN (http://ualcan.path.uab.edu) is a site that provides comprehensive and interactive information. It enables online analysis of different types of tumor data based on the TCGA. The CellMiner (https://discover.nci.nih.gov/cellminer/home.do) is a tool for integrating and studying pharmacological data of 60 tumor cell types.

## Abbreviations

PAQR3: Progestin and AdipoQ Receptor3
TCGA: The Cancer Genome Atlas
NSCLC: Non-small cell lung cancer
BECN1: Coiled-coil myosin-like BCL2-interacting protein
EGFR: Epidermal growth factor receptor
PPAR γ: Peroxisome proliferation-activated receptor γ
BRCA: Breast cancer
PA: Pulmonary adenocarcinoma
SCNA: Somatic cell copy number alterations
OS: Overall survival
DFS: Disease-free survival
PFI: Progression-free interval
DFI: Disease-free interval
CNV: Copy number variations
SNV: Single nucleotide variations
PRAD: Prostate adenocarcinoma
NB: Neuroblastoma
CHOL: Cholangiocarcinoma
ESCA: Esophageal carcinoma
HNSC: Head and neck squamous cell carcinoma
LIHC: Liver hepatocellular carcinoma
LUAD: Lung adenocarcinoma
LUSC: Lung squamous cell carcinoma
KIRC: Kidney renal clear cell carcinoma
KIRP: Kidney renal papillary cell carcinoma
READ: Rectum adenocarcinoma
THCA: Thyroid carcinoma
UCEC: Uterine corpus endometrial carcinoma
DLBC: Lymphoid neoplasm diffuse large B-cell lymphoma
PAAD: Pancreatic adenocarcinoma
PCPG: Pheochromocytoma and paraganglioma
THYM: Thymoma
TGCT: Testicular germ cell tumors
DSS: Disease specific survival
GBMLGG: Glioblastoma and low-grade glioma
LGG: Brain lower grade glioma
KICH: Kidney chromophobe
EAC: Esophageal adenocarcinoma
CESC: Cervical squamous cell carcinoma
SKCM: Skin cutaneous melanoma
BLCA: Bladder urothelial carcinoma
TME: Tumor microenvironment
TGF-β: Transforming growth factor-β
IFN-γ: Interferon-γ
COAD: Colon adenocarcinoma
MESO: Mesothelioma
OV: Ovarian serous cystadenocarcinoma
TMB: Tumor mutation burden
MSI: Microsatellite instability
NEO: Neoantigen
STES: Stomach and esophageal carcinoma
COADREAD: Colon and rectal cancer
STAD: Stomach adenocarcinoma
KIPAN: Pan‐kidney cohort
UVM: Uveal melanoma
SARC: Sarcoma
LAML: Acute myeloid leukemia
DLBCL: Diffuse large B cell lymphoma
